# Chronic obstructive pulmonary disease in sub-Saharan Africa

**DOI:** 10.5588/ijtld.21.0394

**Published:** 2022-03-01

**Authors:** B. I. Awokola, G. A. Amusa, C. P. Jewell, G. Okello, M. Stobrink, L. J. Finney, N. Mohammed, A. Erhart, K. J. Mortimer

**Affiliations:** 1 Centre for Health Informatics, Computing & Statistics (CHICAS), Lancaster Medical School, Lancaster University, Bailrigg, Lancaster, UK; 2 Department of Clinical Sciences, Liverpool School of Tropical Medicine, Liverpool, UK; 3 Medical Research Council Unit The Gambia at the London School of Hygiene & Tropical Medicine, Fajara, The Gambia; 4 Department of Internal Medicine, Jos University Teaching Hospital, Jos, Nigeria; 5 Department of Medicine, University of Jos, Jos, Nigeria; 6 University of Cambridge Institute for Sustainability Leadership, Cambridge, UK; 7 African Centre for Clean Air, Kampala, Uganda; 8 COPD Research Group, Imperial College, London, UK

**Keywords:** COPD, risk factors, sub-Saharan Africa, systematic review, meta-analysis

## Abstract

**BACKGROUND:**

Chronic obstructive pulmonary disease (COPD) is the third leading cause of death worldwide and an important cause of death in sub-Saharan Africa (SSA). We conducted a systematic review and meta-analysis on the prevalence of and risk factors for COPD in SSA.

**METHODS:**

We conducted a protocol-driven systematic literature search in MEDLINE, EMBASE, CINAHL and Global Health, supplemented by a manual search of the abstracts from thoracic conference proceedings from 2017 to 2020. We did a meta-analysis of COPD prevalence and its association with current smoking.

**RESULTS:**

We identified 831 titles, of which 27 were eligible for inclusion in the review and meta-analysis. The population prevalence of COPD ranged from 1.7% to 24.8% (pooled prevalence: 8%, 95% CI 6–11). An increased prevalence of COPD was associated with increasing age, smoking and biomass smoke exposure. The pooled odds ratio for the effect of current smoking (vs. never smoked) on COPD was 2.20 (95% CI 1.62–2.99).

**CONCLUSION:**

COPD causes morbidity and mortality in adults in SSA. Smoking is an important risk factor for COPD in SSA, and this exposure needs to be reduced through the combined efforts of clinicians, researchers and policymakers to address this debilitating and preventable lung disease.

## Abstract (fr)

**CONTEXTE:**

La bronchopneumopathie chronique obstructive (COPD) représente la troisième cause de décès dans le monde et une cause importante de décès en Afrique subsaharienne (SSA). Nous avons réalisé une revue systématique et une méta-analyse de la prévalence et des facteurs de risque de COPD en SSA.

**MÉTHODES:**

Nous avons réalisé une analyse systématique de la littérature déterminée par un protocole, en utilisant les bases de données MEDLINE, EMBASE, CINAHL et Global Health, complétée d’une recherche manuelle des résumés des conférences sur les maladies respiratoires de 2017 à 2020. Nous avons réalisé une méta-analyse de la prévalence de la COPD et de son association avec le tabagisme.

**RÉSULTATS:**

Nous avons identifié 831 titres, dont 27 étaient éligibles à l’inclusion dans la revue et la métaanalyse. La prévalence de la COPD dans la population générale variait de 1,7% à 24,8% (prévalence groupée : 8%, IC 95% 6–11). La prévalence de la COPD augmentait avec l’âge, le tabagisme et l’exposition aux fumées dégagées lors de la combustion de la biomasse. L’odds ratio groupé pour l’effet du tabagisme actuel (vs. personnes n’ayant jamais fumé) sur la COPD était de 2,20 (IC 95% 1,62–2,99).

**CONCLUSION:**

La COPD est à l’origine d’une morbidité et d’une mortalité chez les adultes en SSA. Le tabagisme est un facteur de risque important de COPD en SSA. Cette exposition doit être réduite grâce aux efforts conjoints des cliniciens, chercheurs et responsables politiques afin de s’attaquer à cette maladie pulmonaire évitable et invalidante.

COPD is a progressively debilitating disease, characterised by persistent respiratory symptoms and airflow limitation due to airway abnormalities caused by significant exposure to noxious stimuli, air pollution and poor pre- and post-natal lung growth.^1^,^2^ Once regarded as a disease of high-income countries, COPD is now recognised as a major health problem in low- and middle-income countries (LMICs).^3^–^7^ It is currently the third single cause of mortality worldwide: 3.8 million deaths due to COPD were reported in 2019, with approximately 90% of these occurring in LMICs.^8^–^12^ The WHO estimates that about 251 million people have COPD worldwide and that this burden is increasing.^13^–^15^

Smoking is the main risk factor for COPD.^16^ In LMICs, the use of biomass fuel has been put forward as an important risk factor, but the current evidence to support this assertion is weak.^17^–^19^ Other risk factors include occupational dust exposure and previous serious lung infections such as TB.^2^,^7^,^20^

COPD is often unrecognised by patients and physicians and therefore underdiagnosed and undertreated, especially in many SSA settings, where attention remains more focused on communicable diseases.^21^–^24^ The Gambia, for example, currently lacks population COPD prevalence estimates, the closest study being the Nigerian Burden of Obstructive Lung Disease (BOLD) study, which reported a 7% prevalence among adults in 2015.^25^

We therefore conducted a systematic review and meta-analysis to assess the current prevalence of COPD in SSA and its risk factors. This review is intended to assist clinicians with diagnostic issues and draw the attention of policymakers to strategic interventions on risk factors to reduce the burden of COPD. The current review builds on and updates an earlier systematic review we carried out in 2012 by Finney et al.^26^

## METHODOLOGY

We followed the steps outlined in the PRISMA guidelines.^27^ The search strategy and systematic review protocol was registered with PROSPERO http://www.crd.york.ac.uk/PROSPERO (ID: CRD42019138198).

### Data sources and search strategy

We searched MEDLINE, EMBASE, CINAHL and Global Health in a comprehensive manner using a protocol-driven search strategy (see Supplementary Data 1). We also searched clinical trial registers, reference lists from published reviews and included publications and abstracts from major thoracic medicine conference proceedings of the American Thoracic Society (ATS), the European Respiratory Society (ERS) and proceedings of the Pan African Thoracic Society (PATS) and the International Union Against Tuberculosis and Lung Diseases (The Union) from 2017 to 2020. We searched in English language and no translations were required.

### Study selection and data extraction

Studies were included if they met the following criteria: 1) focus on COPD, its prevalence and risk factors, 2) physician-diagnosed COPD exacerbations, 3) all COPD severities, 4) study population included people from SSA aged ≥18 years, 5) use of spirometry for COPD diagnosis, and 6) any COPD outcome—hospitalisation, discharge or death. Studies on pneumonia in chronic airway diseases, nonhuman subjects, presumptive COPD diagnoses and pharmaceutical agents were excluded. Meeting all of criteria (1), (4) and (5) were critical for inclusion.

Studies were initially assessed for inclusion based on their titles and abstracts. Full texts were obtained for studies potentially meeting the inclusion criteria. Titles, abstracts and full texts were screened for inclusion by two independent reviewers (BA and GA) separately, with each reviewer maintaining a separate record of the data extracted. Data were extracted using a standardised data extraction form that was piloted before the study (see Supplementary Data 2). A third author (CJ) reviewed the output and adjudicated in case of disagreement until a consensus was reached. We retained studies that provided numerical estimates of COPD prevalence in SSA and had clearly defined research methodologies, especially regarding the spirometric diagnosis of COPD. The case definition for COPD had to comply with one of the following: 1) Global Initiative on Obstructive Lung Disease (GOLD) criteria,^28^ or ATS/ERS criteria^29^ (see Supplementary Data 3).

### Quality assessment

The methodological quality of the observational studies was assessed using the Newcastle-Ottawa scale.^30^ A score out of a maximum of six was used for all the selected cross-sectional studies.

### Data analysis

Data were entered into an MS Excel^®^ (Microsoft, Redmond, WA, USA) spreadsheet and then read into RStudio^®^ (Rstudio, Boston, MA, USA).^31^ We used the *metaphor* and *metaviz* packages in R Studio^®^ to establish COPD prevalence and the effect of current smoking on COPD in SSA. We aggregated the population-based studies separately from the occupational-based studies. A random-effects model was used to pool individual studies included in the meta-analysis and to estimate the pooled effect of current smoking (vs. never smoked) on COPD prevalence. We decided not to assess for biomass exposure, as the relevant data were missing in most studies.

### Assessment of heterogeneity

Study heterogeneity was assessed using Cochrane’s *Q*, which was quantified with the inconsistency test (*I*^2^) using a random-effects model in the Cochrane *Q* statistic.^32^ The forest plots were also visually inspected for closeness of point estimates and overlapping confidence intervals (CIs). The accompanying Baujat plot showed the contribution of each study to the observed heterogeneity. Finally, meta-regression was done for identified factors in order to explore the reason for a high heterogeneity when found.

### Publication bias

The risk of publication bias was tested by constructing a funnel plot for a random effects-model.^33^ The extracted study information was the modelling input and a pictorial depiction of how the studies fall within the ‘funnel’ was the output.

## RESULTS

### Study selection

**Figure 1:**
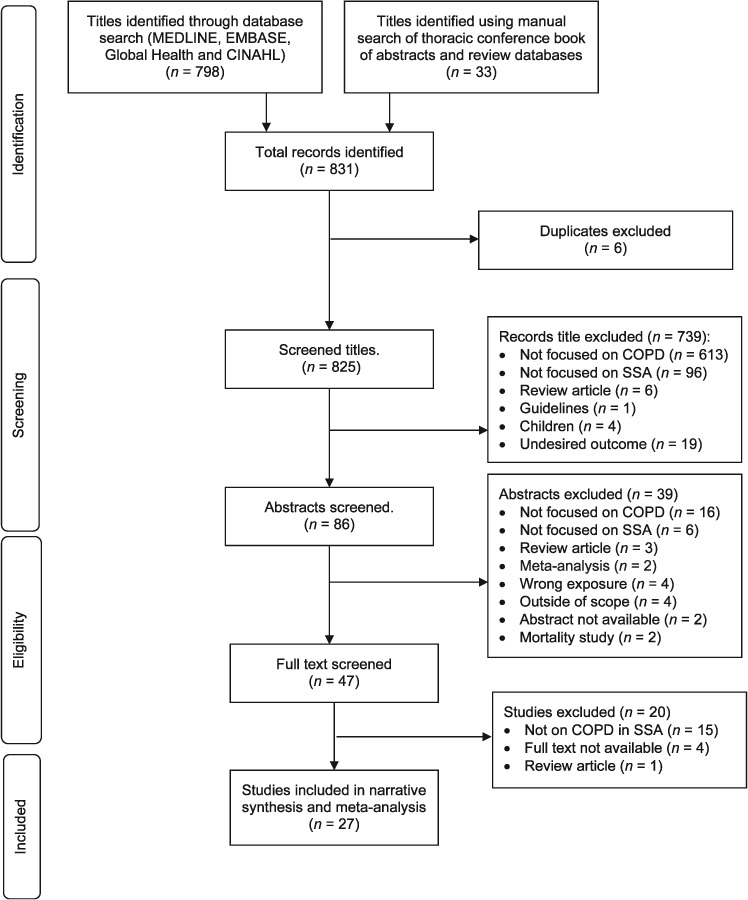
PRISMA flow diagram depicting the selection process. COPD = chronic obstructive pulmonary disease; SSA = sub-Saharan Africa; PRISMA = Preferred Reporting Items for Systematic reviews and Meta-Analyses.

The search yielded a total of 831 titles: MEDLINE (368 titles), EMBASE (222 titles), CINAHL (72 titles), Global Health (136 titles) and 33 titles came for handsearching the book of abstracts for ATS, ERS, PATS and The Union conferences. After the removal of duplicates, 825 titles remained. These were screened for relevance, study location, outcomes of interest and a focus on COPD leaving only 86 abstracts. After excluding mortality studies, out of scope studies, review articles and others not in line with the set protocol, we obtained full text versions of 47 papers for further consideration. Of these, 20 publications were excluded (studies not focused on COPD in SSA, and some were review articles). Twenty-seven studies were then included in the systematic review narrative and the meta-analysis (Figure 1). These comprised 23 population-based studies and four occupational-based studies.

### Study characteristics

All studies included were cross-sectional studies and were conducted between 1977 and 2019 (Table 1).^20^,^25^,^34^–^52^ In total, they represent 17,566 adults, with mean ages ranging between 38 and 80 years. Males and females were usually equally represented in the studies, except two that had industrial settings (i.e., cement and shoe factories), where only males were employed.^50^,^51^

**Table 1:** Overall characteristics of the selected studies on COPD confirmed using spirometry in sub-Saharan Africa

Author	Country	Study period	Participants *n*	Population	Age mean (range) years	COPD definition used	COPD prevalence %	Method quality score
Population-based studies								
Burney^20^	Cape Town, South Africa	2005	840	Urban	M: 53	PBD FEV_1_/FVC <LLN	18.9	5/6
					F: 54			
Buist^34^	Cape Town, South Africa	2007	896	Urban	M: 52.7	PBD FEV_1_/FVC <0.7	24.8	6/6
					F: 54.2 (40–>70)	FEV_1_/FVC<0.7 and FEV_1_ ≤ 80% predicted		
Burney^20^	Gezira, Sudan	2016	575	Rural	M: 55	PBD FEV_1_/FVC <LLN	5.6	5/6
					F: 52			
Burney^20^	Khartoum, Sudan	2014	516	Urban	M: 55	PBD FEV_1_/FVC <LLN	10.5	5/6
					F: 51			
Fullerton^35^	Malawi	2011	372	Mixed	41.53	Pre-BD FEV_1_/FVC <0.7	16	4/6
Garthuru^36^	Nigeria	2002	410	Urban	47.8 (30–69)	Pre-BD FEV_1_/FVC <0.7	9.3	4/6
Burney^20^	Benin	2013	545	Urban	M: 53	PBD FEV_1_/FVC <LLN	7.7	5/6
					F: 50			
Magitta^37^	Tanzania	2016	496	Rural	51.8 (41.2–62.4)	PBD FEV_1_/FVC< 70%	17.5	5/6
Burney^20^	Blantyre, Malawi	2016	401	Urban	M: 53	PBD FEV_1_/FVC <LLN	8.2	5/6
					F: 51			
Burney^20^	Chikhwawa, Malawi	2016	432	Rural	M: 54	PBD FEV_1_/FVC <LLN	14	5/6
					F: 52			
Musafari^38^	Rwanda	2009	1824	Urban and rural	38.3 (15–80)	Pre-BD FEV_1_/FVC <LLN	4.5	5/6
Ngahane^39^	Cameroon	2016	337	Rural	46 (37–59)	PBD FEV_1_/FVC <LLN	18.4	4/6
Nightingale^40^	Malawi	2016	1481	Rural	43.8 (36.0–61.6)	FEV_1_/FVC <0.7	8.7^*^	6/6
North^41^	Uganda	2019	565	Rural	39 ± 17	PBD FEV_1_/FVC < LLN	2.0	6/6
Obaseki^25^	Ile-Ife, Nigeria	2005	1169	Urban	≥40	PBD FEV_1_/FVC < LLN	7.7	6/6
Ozoh^42^	Lagos, Nigeria	2012	412	Urban	53.7 (42.5–64.9)	PBD FEV_1_/FVC < 0.7	5.3	4/6
Pefura-Yone^43^	Cameroon	2014	1287	Urban	34.4 (21.6–47.2)	PBD FEV_1_/FVC < LLN and FEV_1_/FVC <0.7	2.4	5/6
Siddharthan^44^	Uganda	2016	837	Rural	49.1	PBD FEV_1_/FVC *Z*-score ≤–1.64	6.1	5/6
Siddharthan^44^	Uganda	2016	665	Urban	44.1	PBD FEV_1_/FVC *Z*-score ≤–1.64	1.7	5/6
Van Gemert^45^	Uganda	2012	588	Rural	45.0 (31.3–58.7)	FEV_1_/FVC < LLN and FVC <80% as cut-off	16.2	6/6
Wicht^46^*	South Africa	1977	509	Urban	40.2 (median 20–79)	FEV_1_/FVC <0.7	9.3	4/6
Wolderamanuel^47^	Ethiopia	2019	734	Rural	39.15 ± 9.36)	PBD FEV_1_/FVC <0.7	17.8	5/6
Zoller^48^	Tanzania	2016	598	Urban and rural	46.0 (37–57)	FEV_1_/FVC < 5^th^ percentile, FEV_1_ < 0.7	4 (ATS), 5(GOLD)	6/6
Occupational studies								
Girdler-Brown^49^†	South Africa	2008	779	Goldmines	47.8 (30–69)	FEV_1_/FVC <0.7	9.3	4/6
Mbelambela^50^	Democratic Republic Congo	2016	379	Cement factories	Exposed to cement dust: 48 (37.6–58.4)	PBD FEV_1_/FVC < LLN	28.2 (Exposed)	4/6
					Non-exposed: 51.8 (51.7–51.9)	FEV_1_/FVC <70%	9.6 (non-Exposed)	
Oleru & Onyekwe^51^	Nigeria	1992	134	Shoe factory	33.1	FEV_1_/FVC ≤0.7 and FVC ≥0.8 of predicted FVC	6.8	4/6
Rusibamayila^52^	Tanzania	2017	112	Goldmine	37.4 (31.0–43.8)	FEV_1_/FVC < 0.7	1.9	5/6

^*^ Signifies prevalence of obstructive airway disease.

^†^ Underground and open pits.

COPD = chronic obstructive pulmonary disease; M = male; F = female; PBD = post bronchodilator; FEV_1_ = forced expiratory volume in 1 sec; FVC = forced vital capacity; LLN = lower limit of normal.

In all 27 studies, COPD diagnosis was based on spirometry,^20^,^25^,^34^–^52^ mainly based on the GOLD criteria (20 studies), while 6 studies used the ERS/ATS criteria, and 1 study used both criteria. The last study^48^ was analysed alongside the GOLD criteria studies for the sake of computational convenience (Supplementary Data 3).

**Figure 2:**
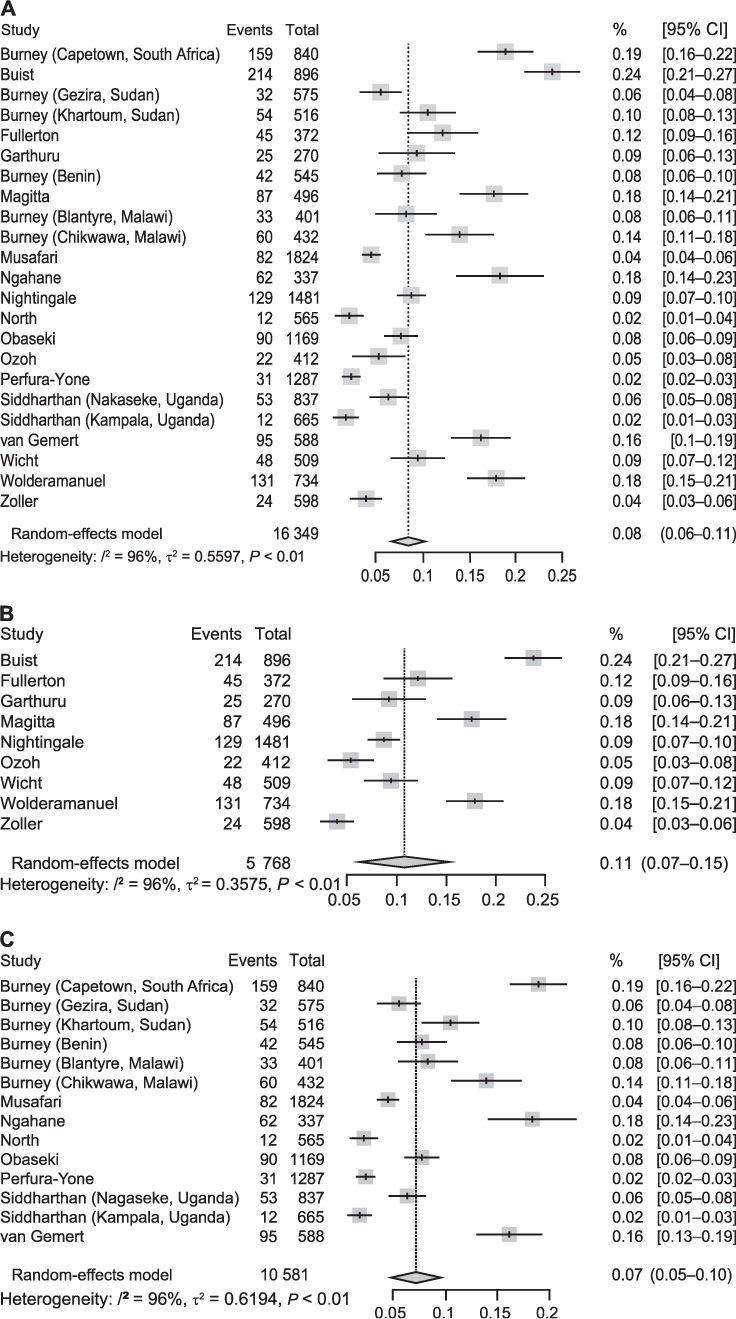
Forest plots showing the study-specific and pooled prevalence of (top to bottom): A) all 23 population-based studies, B) studies using a fixed ratio to define COPD, and C) studies using LLN to define COPD. CI = confidence interval; COPD=chronic obstructive pulmonary disease; LLN = lower limit of normal.

The prevalence of COPD ranged between 1.7% and 24.8% across the population-based studies (Table 1); the pooled prevalence was estimated at 8% (95% CI 6–11) (Figure 2). Prevalence across the occupational-based studies was 1.9% to 28.2% (Table 1), with a pooled prevalence of 9% (95% CI 3–23) (Supplementary Data 4).

The respondents in the studies reported a history of exposure to smoking, biomass fuel and occupational dust; the prevalence of the exposures were documented (Table 2, Supplementary Data 5, Supplementary Data 6). A prior history of TB was also another highlighted risk factor (Table 2). In the studies where information regarding sex and smoking status was provided, significantly more men than women were current smokers (Table 2).

**Table 2:** Exposure to different risk factors for COPD in sub-Saharan Africa in the selected studies ^*^

Study, year	Sex *n* (%)	Smoking status			Biomass exposure %	History of TB	Occupational dust exposure %

		Current %	Ever %	Never %			
Population-based							
Burney, 2016^20^	M: 311 (37.0)	10.0	84.4	76.0	—	19	26.0
	F: 529 (63.0)	6.4	57.9	16.0		12	87.0
Buist, 2007^34^	M: 335 (37.0)	56.9	83.0	17.0	—	19.2	61.9
	F: 561 (63.0)	40.6	59.0	41.0	—	11.9	38.8
Burney, 2016^20^	M: 298 (51.8)	8.0	47.8	68	—	0	18.0
	F: 277 (48.2)	0.2	1.4	56			58.0
Burney, 2014^20^	M: 306 (59.3)	6.4	38.4	44	—	1	90.0
	F: 210 (40.7)	0.8	2.9	14			71.0
Fullerton, 2011^35^	M: 126 (37.9)	7.5	21.4	78.6	100	5.1	—
	F: 206 (62.1)						
Garthuru, 2002^36^	M: 235 (57.2)	9.4	32.2	67.8	—	—	—
	F: 175 (42.8)	0.0	0.6	99.4			
Burney, 2016^20^	M: 237 (43.5)	0.4	4.6	78	—	0	65
	F: 308 (56.5)	0	0	100			96
Magitta, 2016^37^	M: 263 (53.0)	5.4	19.8	74.8	99.5	10.0	—
	F: 233 (47.0)						
Burney, 2016^20^	M: 160 (40.0)	7.5	30.6	38.0	—	9	75.0
	F: 241 (60.0)	0.2	2.5	51.0	—		62.0
Burney, 2016^20^	M: 221 (51.2)	10.9	48.6	58.0	—	7	36.0
	F: 211 (48.8)	2.1	11.3	63.0			84.0
Musafari, 2009^38^	M: 878 (48.1)	20.9	19.8	59.2	5.3	—	20.5
	F: 946 (51.9)	12.6	11.8	75.5			
Ngahane, 2016^39^	M: 168 (49.1)	9.5	—	—	100.0	—	—
	F: 169 (50.9)						
Nightingale, 2013^40^	M: 637 (43.0)	22.2		77.8	99.2	3.2	—
	F: 844 (57.0)						
North, 2019^41^	M: 217 (38.0)	10.0	19.0	71.0	—	—	—
	F: 348 (62.0)						
Obaseki, 2016^25^	M: 609 (40.0)	2.3	8.4	89	67.9	0.5	35.3
	F: 915 (60.0)						
Ozoh, 2012^42^	M: 172 (41.7)	1.5	13.8	84.7	23.1	1.5	24.5
	F: 240 (58.3)						
Pefura-Yone, 2014^43^	M: 619 (48.1)	9.3	6.8	83.9	47.6	1.6	—
	F: 668 (51.9)						
Siddhartan, 2016^44^	M: 380 (45.4)	7.9	—	—	99.6	—	—
	F: 457 (62.0)						
Siddhartan, 2016^44^	M: 318 (47.8)	9.8	—	—	93.6	—	—
	F: 347 (52.2)						
van Gemert, 2015^45^	M: 291 (49.0)	20.7	14.8	64.5	92.9 (indoor)	—	—
	F: 297 (51.0)						
Wicht, 1977^46^	M: 272 (49.9)	—	—	—	—	—	—
	F: 273 (50.1)						
Wolderamauel, 2019^47^	M: 421 (57.4)	9.0	2.7	88.3	—	—	90.0
	F: 213 (42.6)						
Zoller, 2016^48^	M: 310 (52.0)	28.1	—	71.9	85.5 (indoor)	21.9	—
	F: 288 (48.0)						
Occupational studies							
Gridler-Brown, 2008^49^	M: 624 (100)	35.0	61.0	39.0	32.2	—	100.0
Mbelambela, 2016^50^	M: 379 (100.0)	Exposed to cement dust: 56 (25.1)	35 (15.5)	132 (59.2)	—	—	58.8
	F: 0 (0.0)	Non-exposed: 35 (22.8)	25 (16.0)	96 (61.3)			
Oleru & Onyekwere, 1992^51^	M: 134 (100)	10.4	—	89	—	—	100.0
Rusibamayila, 2017^52^	M: 107 (99.5)	—	—	—	—	—	100.0
	F: 5 (4.5)						

^*^ Cells with single proportions represents a combined value for both males and females unless otherwise stated.

COPD = chronic obstructive pulmonary disease; M = male; F = female.

All but 14 of the population-based studies showed that the odds ratio (OR) of developing COPD among current smokers vs. non-smokers was at least 1.15.^34^ Eight studies reported very high respondent exposure to biomass sources. In those with over 99% biomass-exposed population, COPD prevalence ranged between 17% and 18.5%.

Of the 15 studies that documented occupational dust exposure, five reported high proportion of its respondents being exposed; all had urban settings, and three were conducted in industrial settings: a shoe factory and gold mines (Tables 1 and 2).

### Assessment of methodological quality

**Figure 3:**
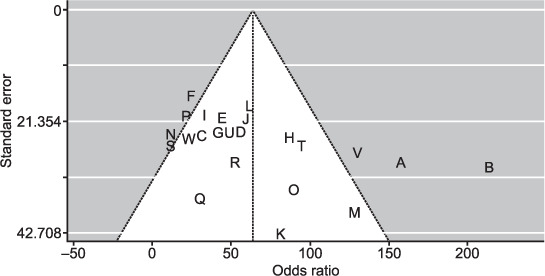
Funnel plot showing the risk of publication bias in the meta-analysis.

The funnel plot for random-effects model for all the population-based studies demonstrated good symmetry. Seventeen out of 23 studies fell within the funnel domain, with three more studies close to the funnel (Figure 3), which indicates a low risk of publication bias. In terms of their Newcastle-Ottawa quality scores, 66% of the studies had a score of at least 5 out of a total of 6, with all studies having a score of at least 4 out of 6 (Table 1).

### Synthesis of results

**Figure 4:**
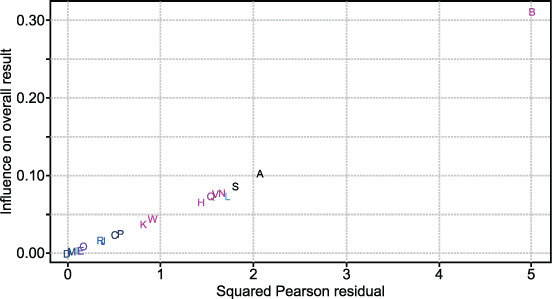
Baujat plot showing the contribution of individual studies to the heterogeneity of the meta-analysis shown in Figure 2. A = Burney et al. (Cape Town, South Africa); B = Buist et al.; C = Burney et al. (Gezira, Sudan); D = Burney et al. (Khartoum, Sudan); E = Fullerton et al.; F = Garthuru et al.; G = Burney et al. (Benin); H = Magitta et al.; I = Burney et al. (Blantyre, Malawi); J = Burney et al. (Chikwawa, Malawi); K =Musafari et al.; L = Ngahane et al.; M = Nightingale et al.; N = North et al.; O = Obaseki et al.; P = Ozoh et al.; Q = Perfura-Yone et al.; R = Siddharthan et al. (Kampala, Uganda [urban]); S = Siddharthan et al. (Nagaseke, Uganda [rural]); T = van Gemert et al.; U = Wicht et al.; V = Wolderamanuel et al.; W = Zoller et al.

**Figure 5:**
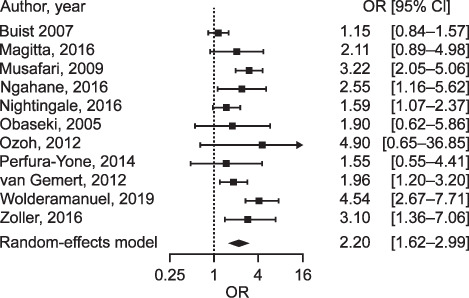
Forest plot displaying the meta-analysis of the effect of current smoking on COPD among respondents from studies on COPD in sub-Saharan Africa that reported current smoking. OR = odds ratio; CI = confidence interval; COPD = chronic obstructive pulmonary disease.

The pooled prevalence of COPD in sub-Saharan Africa from the 23 population-based studies included was 8% (95% CI 6–11; Figure 2). The accompanying Bajaut plot and funnel plots are displayed in Figures 3 and 4, respectively. The pooled OR of current smoking on COPD prevalence is 2.20 (95% CI 1.62–2.99; Figure 5), suggesting that current smoking is a risk for COPD and not a protective factor.

### Heterogeneity assessment

There was considerable heterogeneity between studies (*Q* statistic: 521.13; degrees of freedom: 11; *P* < 0.0001; *I*^2^ = 98%). We eyeballed our data to identify elements that can explain this significant heterogeneity. The definition of COPD (fixed ratio [0.7] vs. lower limit of normal [<5^th^ percentile]), the population studied (rural vs. urban/semi-urban), overall criteria used in study design (GOLD/ERS-ATS), and probably, pre- vs. post-bronchodilator spirometry were possible reasons. We explored these using a sequential meta-regression of all studies on each item, hoping to observe a decrease in the unaccounted variability or *I*^2^ value. The *I*^2^ value did not change much (Supplementary Data 7). Despite this limitation, we proceeded with the meta-analysis to bring together all the data available on COPD prevalence in SSA.

## DISCUSSION

The findings in this systematic review showed that COPD prevalence estimates in sub-Saharan African populations vary widely between studies and settings, from as low as 1.7% (rural Uganda) to as high as 24.8% (urban South Africa), with an average of 8%. African occupational studies had an average COPD prevalence of 9% (range 1.9–28.2). Overall, urban study sites tended to have higher prevalence than rural sites, and COPD prevalence increased with increasing age. As expected, current smoking was the main risk factor for COPD, with a pooled OR of 2.20. Due to the omission of risk factor reporting by many studies, we could not assess the impact of biomass fuel use on COPD prevalence. Our study is the first to utilise a meta-analysis of proportions, alongside a protocol driven systematic analysis, to describe COPD data from SSA.

There have been four previous systematic reviews of COPD in SSA. Three focused on the COPD prevalence in the continent, while one focused on spirometry availability.^22^–^24^,^26^ Mehrotra et al. (2009) reported a wide range of COPD prevalence between different SSA countries and the lack of spirometers and quality spirometry services in much of the subcontinent.^23^ van Gemert et al. (2010) focused on the risk factors for asthma and COPD: current or ever smoking, prior TB, occupational exposures, indoor and outdoor air pollution, biomass fuel use, to mention a few.^22^ The systematic reviews by Adeloye et al. (2012)^24^ and Finney et al. (2013)^26^ highlighted the shortage of spirometry and lack of a noncommunicable disease healthcare strategy in most African countries as barriers to diagnosis.

Similar to the findings in the systematic reviews by Adeloye and van Gemert, we highlight a wide range in COPD prevalence estimates in SSA.^22^,^24^ This may be explained by the variations in the study population (community, primary care clinics, factories, etc.), differences in study settings (urban vs. rural), COPD diagnostic criteria and the effect exerted by other health determinants.

In keeping with findings from Europe, America, Asia and Australasia, we report a rising prevalence of COPD with increasing age.^34^,^53^ The mean age of participants with COPD in the Burden of Obstructive Lung Disease (BOLD) study, involving 12 cities worldwide was 53 years.^20^,^34^ This can be explained by several factors. In recent times, Africa has experienced a demographic shift with increasing life expectancy across many SSA nations. This has been attributed to increased standard of living and improved health-seeking behaviour.^54^ The effect of this is an increase in the number of elderly people, and thus, an increase in the number of cases of COPD. Furthermore, urbanisation and westernisation of African communities have also led to increased prevalence of non-communicable diseases.^5^

Smoking is an established risk factor for developing COPD.^55^–^60^ There was a consensus between our review and others regarding this risk factor.^22^,^24^,^26^ Our meta-analysis showed that studies where two-fifths or more of its participants smoked tobacco, the COPD prevalence was at least 20%. Smoking rates in LMICs have increased as economies develop,^1^,^60^ putting the African continent on the verge of a smoking epidemic.^24^,^60^ Current smoking was highlighted due to availability of data on it in all the selected studies. Selective reporting in many of the retained studies made it difficult for us to conclude on other known risk factors such as biomass exposure, occupational dust exposure and a prior history of TB.

The inclusion of a meta-analysis of the prevalence of COPD in SSA and that of the effect of current smoking on COPD prevalence is a major strength of this systematic review. However, in the light of the unexplained high heterogeneity, this needs to be interpreted carefully. Second, the assessment of risk factors for COPD was another strength. Third, 13 countries were represented in this review compared to four in the previous one by Finney et al.,^26^ which makes our results more generalisable. Fourth, to ensure that we have good confidence in our conclusions, only studies with spirometry-based diagnoses were included. Finally, all the 27 spirometry-based studies used currently acceptable case definition criteria in diagnosing the participants: either ERS-ATS or the GOLD criteria. This is also an improvement over the systematic review conducted by Finney et al., where only six spirometry-based studies used up-to-date criteria, with eight other studies using spirometry without strictly following the standard guidelines.^26^

There was a high level of heterogeneity in the studies selected but we decided to execute the meta-analysis to bring together all the data available on COPD prevalence in SSA following an unsuccessful exploration of this heterogeneity using meta-regression analysis. We acknowledge this is a study limitation. Also, in a bid to capture as much spirometry-diagnosed COPD cases in our systematic review, we included three population-based studies that used pre-bronchodilatory FEV_1_/FVC (forced expiratory volume in 1 sec/forced vital capacity) ratio in defining COPD.^35^,^36^,^38^ This may have inadvertently led to the inclusion of patients with bronchial asthma, along with COPD. The absence of complete information about other risk factors besides smoking is another limitation, especially in SSA settings, where other risk factors in addition to smoking exist. This could have been an opportunity for collecting evidence, given that current evidence supporting biomass fuel as a risk factor for developing obstructive lung disease is weak.^17^–^19^,^35^

The need to identify the true burden of COPD in SSA cannot be overemphasised. This will provide stakeholders with a clearer perspective on the magnitude of this neglected health problem, which should enable them to respond appropriately. Accurate quantification of the burden of COPD using spirometry is needed now more than ever. Spirometry is currently unavailable in many parts of SSA, thus hampering objective diagnosis as a prelude to effective therapy.^23^,^61^,^62^ Mehrotra et al. reported an availability of spirometry service and spirometer for COPD diagnosis among less than 20% of physicians contacted in 34 African countries.^23^ Where these were available, spirometry results were of variable quality, based on the skills of the operator.^40^,^61^ In addition to providing access to spirometers, health workers using spirometry need proper training and re-training. Health policy makers need to ensure high-quality spirometry and spirometers in the existing public health service delivery channels to improve the diagnosis and management of chronic lung diseases.

In order to improve the awareness, diagnosis and management of COPD in SSA, clinical and operational research is crucial. Regarding COPD research in SSA, a lot still needs to be done to quantify the problems, proffer sustainable solutions and drive policy change successfully.^61^,^63^ Awareness campaigns on the dangers of tobacco use, tobacco use control and anti-tobacco legislation are additional vital steps to be taken for any government interested in curtailing the increase in COPD prevalence. Clinicians should also inquire about the willingness to quit smoking among smokers and be able to provide basic tobacco cessation services.^22^ Unfortunately, many governments in SSA have not yet given COPD the attention it requires. Concerted efforts are thus needed to ensure that this untreatable, yet potentially preventable disease is put on the health agenda of LMICs where the impact is most felt.

In a bid to control COPD, tobacco use control, anti-tobacco awareness campaigns and anti-tobacco legislations must be taken very seriously and pushed aggressively by all concerned. While the following were not shown in this review, biomass exposure, occupational dust exposure and traffic-related exposure should also be reduced in every country. Establishing and enforcing these and other similar initiatives will likely deliver lung health benefits in good time, thus bringing the menace of COPD in SSA under good control.

In conclusion, COPD causes a substantial burden of morbidity and mortality in SSA. The ongoing demographic shift in SSA suggests that this burden will increase in line with increases in life expectancy. Smoking and biomass fuel exposures are important risk factors for COPD in SSA and these exposures need to be substantially reduced through the combined efforts of clinicians, researchers and policymakers.
